# Novel Chimeric Vaccine Candidate Development against *Leptotrichia buccalis*

**DOI:** 10.3390/ijerph191710742

**Published:** 2022-08-29

**Authors:** Abdulrahman Alshammari, Abdullah F. Alasmari, Metab Alharbi, Nemat Ali, Ziyad Tariq Muhseen, Usman Ali Ashfaq, Miraj Ud-din, Asad Ullah, Muhammad Arshad, Sajjad Ahmad

**Affiliations:** 1 Department of Pharmacology and Toxicology, College of Pharmacy, King Saud University, P.O. Box 2455, Riyadh 11451, Saudi Arabia; 2 Department of Pharmacy, Al-Mustaqbal University College, Hillah, Babylon 51001, Iraq; 3 Department of Bioinformatics and Biotechnology, Government College, University Faisalabad, Faisalabad 38000, Pakistan; 4 Department of Health and Biological Sciences, Abasyn University, Peshawar 25000, Pakistan; 5 Centre of Biotechnology and Microbiology, University of Peshawar, Peshawar 25000, Pakistan; 6 Department of Computer Science, Virginia Tech, Blacksburg, VA 24061, USA

**Keywords:** *Leptotrichia buccalis*, multi-epitopes vaccine, molecular docking, molecular dynamics simulation

## Abstract

The misuse of antibiotics in our daily lives has led to the emergence of antimicrobial resistance. As a result, many antibiotics are becoming ineffective. This phenomenon is linked with high rates of mortality and morbidity. Therefore, new approaches are required to address this major health issue. *Leptotrichia buccalis* is a Gram-negative, rod-shaped bacterium which normally resides in the oral and vaginal cavities. It is an emerging bacterial pathogen which is developing new antibiotic-resistance mechanisms. No approved vaccine is available against this pathogen, which is a cause for growing concern. In this study, an in silico-based, multi-epitopes vaccine against this pathogen was designed by applying reverse vaccinology and immunoinformatic approaches. Of a total of 2193 predicted proteins, 294 were found to be redundant while 1899 were non-redundant. Among the non-redundant proteins, 6 were predicted to be present in the extracellular region, 12 in the periplasmic region and 23 in the outer-membrane region. Three proteins (trypsin-like peptidase domain-containing protein, sel1 repeat family protein and TrbI/VirB10 family protein) were predicted to be virulent and potential subunit vaccine targets. In the epitopes prediction phase, the three proteins were subjected to B- and T-cell epitope mapping; 19 epitopes were used for vaccine design. The vaccine construct was docked with MHC-I, MHC-II and TLR-4 immune receptors and only the top-ranked complex (based on global energy value) was selected in each case. The selected docked complexes were examined in a molecular dynamic simulation and binding free energies analysis in order to assess their intermolecular stability. It was observed that the vaccine binding mode with receptors was stable and that the system presented stable dynamics. The net binding free energy of complexes was in the range of −300 to −500 kcal/mol, indicating the formation of stable complexes. In conclusion, the data reported herein might help vaccinologists to formulate a chimeric vaccine against the aforementioned target pathogen.

## 1. Introduction

The human body is a good host for many microorganisms. The majority are part of the normal flora, while others are pathogens that cause various types of acute and chronic infections [1]. *L. buccalis* is a Gram-negative, anaerobic and rod-shaped bacterium that is mostly found in human oral cavity, intestine and also the female genitalia [1]. *Leptotrichia* species are typically large, fusiform-shaped, non-sporulating and non-motile and are found as part of oral biofilms. The genus *Leptotrichia* was described by Trevisan as including numerous filamentous species. Severe infections involving *L. buccalis* have been reported in the literature [2]. Some of the important infectious diseases caused by *L. buccails* are bacteremia, endocarditis and hepatic abscess [3]. *Leptotrichia* species have been reported to harbor novel resistance genes, and it is hypothesized that they will become more powerful in the future, with potential for severe outbreaks [4]. One contributing factor in the dissemination of these bacteria is the discharge of rural and urban waste water into the sea water and its mixing with drinking water sources. This allows the bacteria to spread very fast in the community. Therefore, serious efforts are needed to stop the spread of *Leptotrichia* species in the environment.

Numerous microorganisms have developed antibiotic resistance, and the spread of new antibiotic-resistant bacterial strains will place a significant burden on healthcare systems [5]. There are many ways to tackle this global issue. It can be controlled by developing more powerful, novel classes of antibiotics, by stopping the misuse of antibiotics or by developing vaccines. Vaccine development is an effective way to tackle bacterial infections [6]. Vaccines are biological preparations which are capable of inducing adaptive immunity against pathogens [7,8]. Vaccines are one of the most important inventions in the field of medicine. The successful development of vaccines has contributed to overcoming and eradicating several infectious diseases. One such example is smallpox, which has killed millions of people. However, with the development of a vaccine, morbidity from the disease was reduced from 2 million cases per year to zero [9]. The success of other vaccines, for example against polio, meningitis and pneumococcus, has revealed the importance of such an approach [10]. Vaccines are effective against many pathogens but are not sufficient against others, as they cannot be cultivated [11].

Most vaccines prior to 1980 targeted viruses, but knowledge about the conjugation of bacterial capsular polysaccharides to proteins has led to the development of vaccines against *Haemophilus influenza Type B*, *Meningococcus* and *Pneumococcus* [12]. This was made possible by the introduction of new techniques, especially reverse vaccinology. Reverse vaccinology is a genome-based approach toward vaccine development in which there is no need to grow the pathogen [13,14]. In this approach, the pathogen genome is screened for possible protective antigens considering several candidate vaccine filters [15,16]. Considering the widespread efforts to develop a vaccine against *L. buccalis*, the bacterial genome is available in public databases. This provides opportunities to thoroughly screen the pathogen in order to identify protective antigens and formulate the epitopes for a chimeric vaccine. Several analyses have been performed to accomplish this objective. The findings are expected to speed up vaccine design against *L. buccalis* and save large amounts of money in the experimental identification of antigens.

## 2. Research Methodology

### 2.1. Complete Genome Retrieval

The complete sequenced genome of *L. buccalis* was retrieved from the NCBI database with RefSeq accession id GCF_000023905; the data comprised 2.466 MB in size. The complete genome is fully annotated and was therefore selected for investigation. The retrieved genome was then examined on a CD–HIT server [12] to remove redundant proteins at a threshold of 50%. The non-redundant proteins were selected for further studies. Redundant proteins are duplicated sequences while non-redundant proteins are single representations in the complete genome [17].

### 2.2. Subcellular Localization and Virulent Protein Analysis

The subcellular localization of non-redundant proteins was done using the PSORTb server [13]. The proteins were used as an input file and those present on the surface of the pathogen, i.e., in outer membrane, periplasm or extracellular, were shortlisted. These proteins are exposed to the host immune system and contain antigenic regions, which are useful when designing a vaccine [18]. The PSORTb results were validated using the CELLO2Go software [19]. The short-listed proteins were further investigated in a virulent protein analysis using the virulent factor database (VFDB) [20]. Virulent proteins initiate infectious pathways and are more immunogenic [11]. The selection criteria of virulent proteins were sequence similarity ≥30% and bit score ≥100 [21].

### 2.3. Epitope Prediction

The immune epitopes database (IEDB) server was used for the prediction of epitopes for the selected proteins [14]. First, linear B-cell epitopes were predicted and then used for the prediction of T cell epitopes [15]. B-cell epitope prediction was done using Bepipred linear epitope prediction 2.0 [22] with a cut-off value of 0.5. During the prediction of T-cell epitopes, MHC-I epitopes were first predicted followed by the MHC-II epitopes. The selection of the epitopes was made on the basis of the percentile score, i.e., epitopes with low percentile scores were selected [16]. To make the selection, a comparative analysis was done and only common epitopes were chosen. In both MHC-I and MHC-II epitopes, a reference set of MHC alleles was used, as given on the IEDB server.

### 2.4. Epitopes Prioritization Phase

The different properties of the epitopes were predicted in order to shortlist only those which would be suitable for chimeric vaccine design. The antigenicity of the epitopes was determined using the VaxiJen tool v2.0 [23], while the allergenicity was predicted using the ALLERTOP tool v2.0 [24]. The water solubility of the epitopes was evaluated using the Innovagen peptide solubility calculator of (https://pepcalc.com/peptide-solubility-calculator.php (accessed on 3 April 2022)). The toxicity of the epitopes was checked using the ToxinPred server [25]. Probable antigenic, non-allergenic, water soluble and non-toxic epitopes were shortlisted for further processing.

### 2.5. Vaccine Construct Design

The issue with single epitope vaccines is that they do not induce good immune responses. To overcome this problem, a multi-epitopes vaccine was designed to generate a stronger immune response [26]. After the analysis of epitopes, suitable epitopes were selected to design a multi-epitope vaccine construct. GPGPG linkers were used to attach the epitopes together. The epitope peptide was joined at the N-terminal with the cholera toxin B subunit, which has been shown to be a safe and effective adjuvant [27]. The epitope peptide linkage with the adjuvant was done using the EAAAK linker. Both the GPGPG and EAAAK linkers ensured the domains remained at distance and allowed them to be effectively presented to the host immune cells [28].

### 2.6. Structure Modelling of the Vaccine Construct

The 3D structure of the final vaccine was devised using 3D pro server [29]. Due to the non-availability of an appropriate template for modeling, ab initio modeling was used (homology modeling was not desirable). Several structures were suggested by the server and the best one was selected. Some loops were modelled for the final vaccine construct using the Galaxy web server [30]. The loop refined structure was then investigated for structure refinement using the same technology [31]. Loop modelling and refinement was done to confer stability to the vaccine and to lower the global energy of the vaccine.

### 2.7. Disulfide Engineering

Disulfide engineering is important for the conformational stability of folded proteins [26]. The disulfide engineering of the vaccine was performed using an online server called Disulfide by Design 2.0 [32]. Thanks to this server, pairs of residues that had high energy values and were prone to enzymatic degradation were replaced by cysteine residues.

### 2.8. Molecular Docking

The vaccine structure was then processed on a PATCHDOCK server [33] for blind docking studies with different immune receptors. The structures of the MHC-I, MHC-II and TLR-4 receptors were downloaded from the protein data bank using pdb id 1L1Y, 1KGO and 4G8A, respectively. For each vaccine and receptor, 20 solutions were generated; these were consequently refined for structure errors using FireDock software [34]. The top-10 complexes were retrieved and only the best docked complex based on global binding energy was chosen for visualization in UCSF Chimera v.1.15 [35].

### 2.9. Molecular Dynamic Simulation

A molecular dynamics simulation was performed to study the docked dynamics of the designed vaccine with immune receptors [36]. In molecular dynamics simulations, the stability of vaccines may be determined by analyzing the conformational changes in the vaccine immune receptors. Molecular dynamics studies were performed using the AMBER20 simulation package [37]. Initial libraries of the complexes were generated using the Antechamber software [38], while Ff14Sb force filed was used to parametrize the complexes [39]. The simulation protocol was done in four phases, i.e., energy minimization, heating, equilibration and a production run of 150 ns [40]. The stability was evaluated in three different analyses, i.e., root mean square deviation (RMSD) [41], root mean square fluctuation (RMSF) [42] and hydrogen bonds [43]. For plotting, XMGRACE v5.1 was used [44]. The different binding free energies of the complexes were determined using the MMPBSA.py module in AMBER [45,46].

## 3. Results and Discussion

### 3.1. Complete Sequenced Genome Retrieval

The present research was initiated by retrieving the complete sequenced genome of *L. buccalis* from the NCBI genome database. The size of the data was 2.466 MB. The whole sequence consists of total 2193 predicted proteins encoded by 2231 genes. The GC content of the genome is 20.8%. It contains 15 rRNA, 46 tRNA, 4 other RNA and 73 pseudogenes. Only reference proteome were selected for the investigation of good vaccine targets due to their complete functional annotations. The reference proteome also guides the construction of new strains of proteome and supports quick and cheap assembly [47].

### 3.2. CD-HIT Analysis

The predicted *L. buccalis* proteins underwent CD-HIT analysis to remove redundant proteins. Non-redundant proteins are part of a pathogen’s functionality, and are therefore regarded as good vaccine candidates [48]. A total of 294 redundant sequences were removed, while 1899 non-redundant proteins were selected for further studies.

### 3.3. Subcellular Localization

The non-redundant proteins were than checked for their subcellular localization. These proteins are considered to be good vaccine candidates. Surface proteins are in frequent contact with the host’s immune system cells and play a major role in bacterial virulence [18]. These proteins harbor antigenic determinants which are capable of eliciting strong immune reactions [49]. The considered were present in the outer membrane, periplasmic and extracellular matrix. In total, the existence of 23 proteins was predicted in outer-membrane region, 6 extracellular and 12 proteins in the periplasmic membrane region.

### 3.4. Virulent Factor Database Analysis

Virulent proteins are usually considered to be the best vaccine candidates because they have the ability to generate a host immune response [50]. After the VFDB analysis, three proteins, i.e., one outer membrane protein, one periplasmic membrane protein and one extracellular protein, were predicted to be virulent.

### 3.5. B- and T-Cell Epitope Prediction

For epitope predictions, three proteins, i.e., trypsin-like peptidase domain-containing protein, sel1 repeat family protein and TrbI/VirB10 family, were selected. Epitope predictions were performed using the Immune Epitope Debase (IEDB) server. Initially, linear B-cell epitopes were identified. Epitopes are a part of antigens that stimulates both humoral and cellular immunity. In total, five, six and four B-cell epitopes were predicted for trypsin-like peptidase domain-containing protein, sel1 repeat family protein and TrbI/VirB10 family protein, respectively, as shown in Table 1. The B-cell epitopes are key in activating humoral immunity and binding to antibodies [51,52]. The B-cell epitopes were subsequently examined for T-cell epitopes.

In the T-cell epitope prediction, both MHC-II and MHC-II epitopes were predicted. The predicted epitopes were prioritized on the basis of their least percentile score; see Table 2. The T-cell epitopes were coated in MHC molecules and are recognized by T-cell receptors [53]. T-cell epitopes are mainly involved in generating host cellular immune responses against pathogens [28].

### 3.6. Epitope Properties Analyses

Different properties of the epitopes were checked to select the best ones for our chimeric vaccine design. The antigenic and non-allergenic epitopes were further analyzed for their toxicity and water solubility. Antigenic epitopes ensure that antibodies are generated, while non-allergenic epitopes do not give rise to allergic reactions. All epitopes which were antigenic, non-allergenic, toxic and water soluble were selected for our vaccine construct design.

### 3.7. Vaccine Construct Design

**Figure 1:**
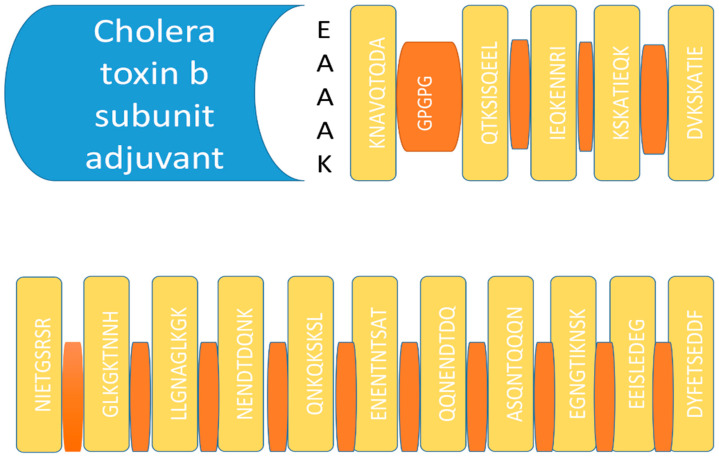
Schematic diagram of a multi-epitope vaccine construct. The blue colored box represents the adjuvant, EAAAK is indicated in black text, epitopes are shown in yellow and GPGPG linkers are shown in orange.

A single epitope vaccine generates very little immune response; therefore, in this study, a multi-epitope vaccine was designed. After analyzing the epitopes, a total of 16 were selected for our multi-epitope vaccine. The vaccine construct was designed by linking the selected epitopes with each other using GPGPG linkers, as presented in Figure 1 [39]. These linkers were used to avoid folding the epitopes over one another and to keep them separated from one another [54]. The separated epitopes have a good chance of coming into contact with the host immune system, thereby generating a robust immune responses.

### 3.8. D Structure, Loop Modelling and Refinement

The 3D structure for the vaccine construct was predicted using ab initio metho, as no appropriate template was found. The vaccine structure was required to evaluate its potential to generate an immune response. The loops have no fixed 3D structure, and were therefore modeled. A total of six loops were modelled using another online tool. i.e., Galaxy web. Vaccine structure refinement was done to remove steric clashes. The refined vaccine had an improved global energy of −1245.02 kcal/mol and good Rama favored regions residues, i.e., 92%.

### 3.9. Disulfide Engineering

**Figure 2:**
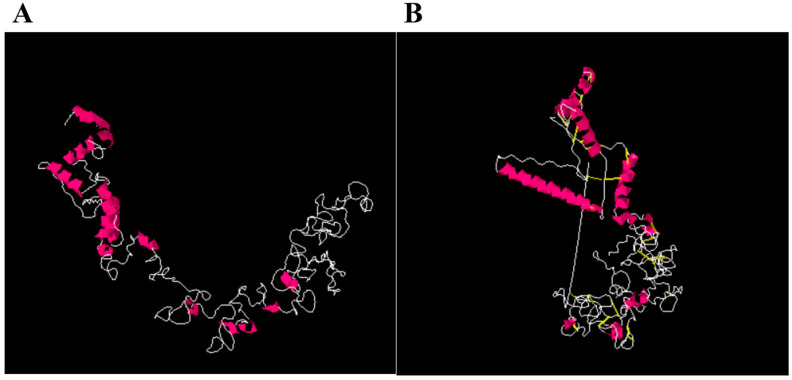
Disulfide engineering of the designed chimeric vaccine. (**A**) Wild-type structure of the vaccine construct. (**B**) Mutated structure of the vaccine construct. Mutated amino acid residues are shown in yellow.

The vaccine was subjected to disulfide engineering to enhance its intermolecular bonds and structural stability. Additionally, this process ensures that weaker components are resistant to cellular degradation [55]. Only groups with a an energy level >0 kcal/mol transform cysteine residues into cysteine. A total of 26 residue pairs in the vaccine structure were found to mutate to cysteine. The cysteine linkages can be seen as yellow sticks because they replaced the amino acid residues, as shown in Figure 2. The residue pairs that were mutated are tabulated in Table 3. It was determined that the vaccine antigenicity value was 0.8.

### 3.10. Molecular Docking

Vaccines need to interact well with immune receptors in order to induce a good immune response. It is vital to understand such interactions, as they can activate many immunological pathways to clear the pathogen. In total, 20 solutions for each docking were retrieved and ordered according to their docking score (Tables S1–S3). The solutions were further refined with the aim of eliminating false positives and selecting the lowest binding energy complexes. Vaccines with the lowest binding energy are more likely to bind strongly to immune receptors. Due to its global energy of −12.7 KJ m^−1^, solution number 6 was selected for MHC-I. In MHC-II, solution number 9 had a global binding energy of −0.17 KJ m^−1^. The lowest energy solution of solution 8 was selected for TLR-4. TLR-4, as a member of the toll-like receptor family, plays a significant role in recognizing pathogen-associated molecular patterns (PAMPs) and, hence, the activation of innate immunological responses. PAMPs are expressed on the pathogen surface and, once recognized, activate cytokine production, which is key for effective immunity development. Table 4, Table 5 and Table 6 show the docked solutions with MHC-I, MHC-II and TLR-4, respectively, as recorded by FireDock.

**Figure 3:**
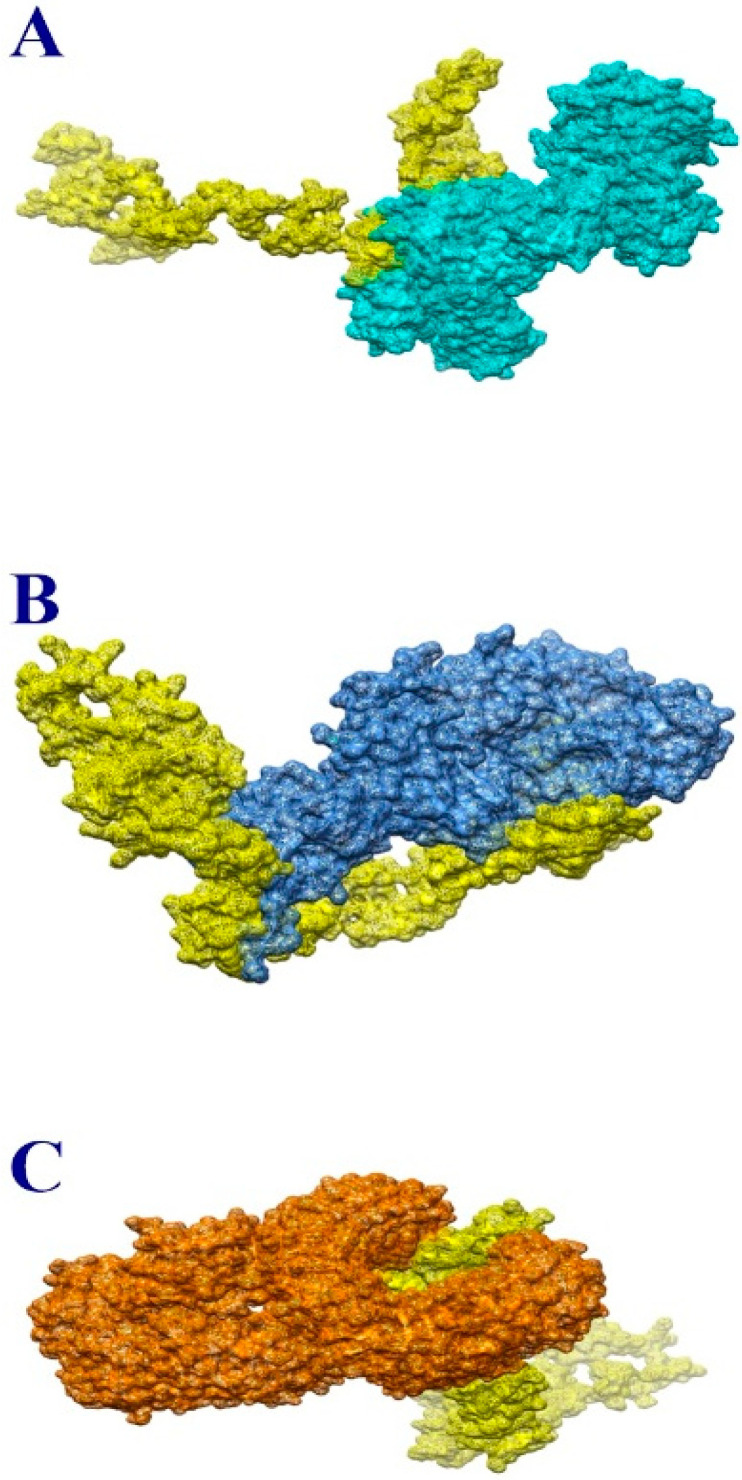
Docked complex of vaccine and MHC-I molecule (**A**), MHC-II molecule (**B**) and TLR-4 (**C**). The vaccine is shown in yellow while MHC-I, MHC-II and TLR-4 are shown in light blue, medium blue and orange, respectively.

The optimal docked complex for each receptor was visualized to evaluate the docked binding mode with immunological receptors, as given in Figure 3A–C. It was revealed that the vaccine was able to bind strongly to the receptors, and the epitopes were exposed to host immune cells. This information implies that epitopes in vaccines can activate powerful immune signaling pathways and elicit significant protective immune responses.

A precise description of the nature and frequency of vaccine–receptor interactions is essential, since these determine the intensity of such interactions. There are several forms of interactions between vaccination and receptors, including hydrophilic, salt bridges, hydrophobic and disulfide bonds. In order to keep the vaccine docked to immunological receptors, all these proteins are crucial. As a result, these interactions require a high number of receptor residues to bind to vaccine components. The residues are listed in Table 7.

### 3.11. Molecular Dynamic Simulation

**Figure 4:**
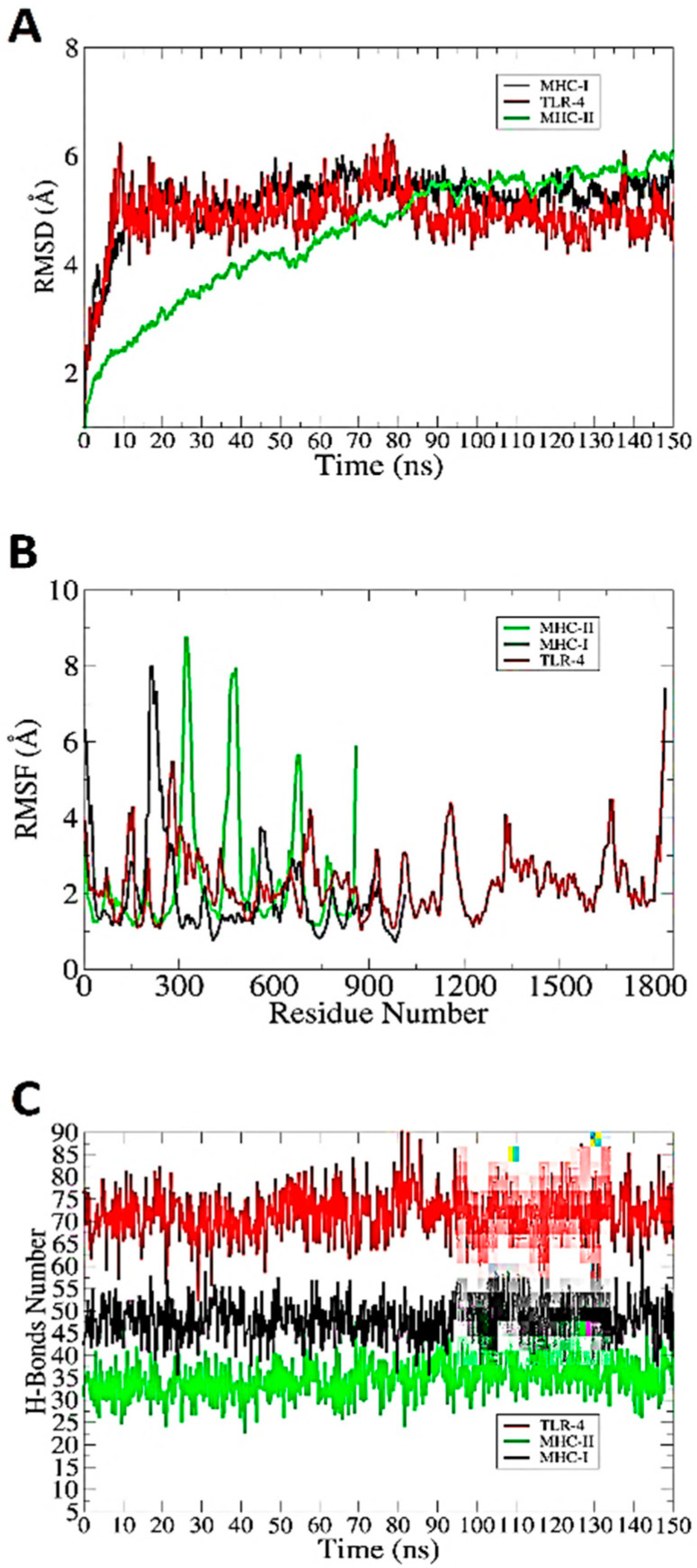
Analysis of simulation trajectories using RMSD (**A**), RMSF (**B**) and (**C**) hydrogen bonds.

The docked complexes were further subjected to molecular dynamics simulations to examine their dynamics. RMSD, RMSF and hydrogen bonding were used to study the simulation trajectories based on carbon alpha atoms. This investigation was essential to understand the binding stability of the vaccine to receptors and to determine whether the vaccine epitopes were exposed to the host immune cells. No significant structural differences in the RMSD plot over the course of the simulated time were noticed. The RMSD, plotted in Figure 4A, fluctuated between 4 and 6 Å during the simulation run. The RMSF results further revealed that the key receptor residues remained stability, with only a few high flexibilities due to the loop regions. The majority of the system residues were below 5 Å; see Figure 4B. The secondary structures were found to have tight conformations, and the systems remained compact during the simulation. The hydrogen bond analysis between the vaccine and immune receptors is presented in Figure 4C. All H-bonds were close, and a good number of hydrogen bonds were found in the vaccine between the MHC-I, MHC-II and TLR-4 receptors. In conclusion, the overall simulation results indicated that there was reasonable stability between the vaccine and immune receptors.

### 3.12. Estimation of Binding Free Energy

In order to calculate the binding free energy of the docked complexes, the MM-GBSA and MM-PBSA techniques were used [47]. Both techniques are extremely fast and highly efficient. In MM-GBSA, the total binding free energy for vaccine-TLR-4 complex was −516.79 kcal/mol; for vaccine-MHC-I complex, it was −399.79 kcal/mol and for vaccine-MHC-II complex −359.37 kcal/mol, as shown in Table 8. In the case of MM-PBSA, the values were −515.77 kcal/mol for vaccine-TLR-4, −402.43 kcal/mol for the MHC-I-vaccine complex and −356.2 kcal/mol for the MHC-II-vaccine complex. Van der Waals and electrostatic energies play an important role in complexation.

## 4. Conclusions and Limitations

Several computer-aided vaccine design approaches have been developed to facilitate the identification of potential vaccine targets [56,57,58,59]. This genome-based approach has been shown to be successful in fast tracking experimental vaccine development and saving time and money [60]. *L. buccalis* is a causative agent of multiple hospital infections, and thus poses a global health threat. In this study, three proteins (trypsin-like peptidase domain-containing protein, sel1 repeat family protein and TrbI/VirB10 family protein) were identified as good subunit vaccine candidates to be examined for their immune protection efficacy against this pathogen. Potential B- and T-cell epitopes were mapped for the aforementioned vaccine targets and used in a multi-epitope peptide construct. The chimeric vaccine showed strong and stable binding with the selected immune receptors. Thus, it is concluded that this vaccine is promising and should undergo experimental testing. However, some limitations in the present study must be considered in future studies. For example, the experimental evaluation of the ordering of epitopes in the vaccine construct must be validated. Also, the predicted epitope antigenicity and immunogenicity must be examined to confirm the predictions in this paper. While this study lacks experimental validation; its findings may speed up vaccine development against *L. buccalis.*

## Figures and Tables

**Table 1:** Predicted B-cells epitopes for shortlisted subunit proteins.

Proteins	B-Cell Epitopes
WP_012806254.1 (trypsin-like peptidase domain-containing protein)	NFMKKGNKKFALF
	KNDTKSNSTENMANVEQTKSISQEELQKYTKNAVQTQDA
	KTVTVNTYNPLEEMLFGRSGGQEKRESGS
	RSSLGIEQI
	TPNALQQQQIIQQRQQQQQQE
WP_015769552.1 (sel1 repeat family protein)	DVKSKATIEQKENNRIK
	GIDTKIDYKKAMEW
	GFGVKKDYKQ
	EKGLGVEKSFDS
	EMAGDYAKA
	GKGVKKNLKEASE
WP_015769221.1 (TrbI/VirB10 family protein)	NDYFETSEDDFTEQKEEEISLEDEGNGTIKNSK
	KKNMKSNEQEKVDSISTGTELDINDAVNTQANKNPQVSETIAQSGTENINTASDKTGTPNLSQYDSQLGSDYNNDNFDSSYGASTSPPSFSNVSENENTNTSATSVAPSEKYKEWRKSSIGFDKGVSTQTPQVPEQYQEQQPASQNTQQQNENDTDQNKQKSKSLFLKQKQDSFYSTNLKNPAIGKYELK
	QGVDLLGNAGLKGKTNNH
	EGLNVNIETGSRSRVNIGTG

**Table 2:** MHC-I and MHC-II predicted epitopes with percentile scores.

MHC-I	Percentile SCORE	MHC-II	Percentile Score
FMKKGNKKF	0.06	FMKKGNKKFAL	2.2
MKKGNKKFAL	0.73	VEQTKSISQEE	2
QTKSISQEE	3.3	KNDTKSNSTENMANV	26
VEQTKSISQ	2.5	ELQKYTKNAVQTQDA	6.1
NSTENMANV	0.13		
KNDTKSNST	18	VEQTKSISQEEL	2.5
LQKYTKNAV	1.1	KNDTKSNSTENMANV	26
KNAVQTQDA	16	ELQKYTKNAVQTQDA	6.1
QTKSISQEEL	0.91		
VEQTKSISQ	2.5	LQQQQIIQQRQQQQ	0.13
NSTENMANV	0.13	TPNALQQQQIIQQRQ	0.38
KNDTKSNST	18		
LQKYTKNAV	1.1	TIEQKENNRIK	3.6
KNAVQTQDA	16	DVKSKATIEQK	4
QQQQIIQQR	0.11		
IIQQRQQQQ	9.1	IDTKIDYKKAM	5.4
NALQQQQII	0.08	IDTKIDYKKAMEW	11
TPNALQQQQI	0.94		
QQQQIIQQRQ	5	KGLGVEKSFDS	13
IEQKENNRI	0.39		
KSKATIEQK	0.01	KGVKKNLKEAS	6.4
DVKSKATIE	4.3		
DTKIDYKKAM	0.52	DYFETSEDDFT	0.62
KIDYKKAMEW	0.07	EQKEEEISLEDEG	0.89
IDTKIDYKK	6	EDEGNGTIKNSK	18
KGLGVEKSF	0.48		
LGVEKSFDS	45	ASQNTQQQNENDTDQ	100
GVKKNLKEA	0.78	ASTSPPSFSNVSENE	100
KGVKKNLKEA	1.4	ENENTNTSATSVAPS	97
DYFETSEDDF	0.61	KKNMKSNEQEKVDSI	93
EQKEEEISL	0.25	STNLKNPAIGKYELK	92
EEISLEDEG	4.8	KSKSLFLKQKQDSFY	95
EGNGTIKNSK	1.5	NENDTDQNKQKSKSL	95
EDEGNGTIK	11	NENDTDQNKQKSKSL	100
ASQNTQQQN	5.7		
QQNENDTDQ	15	GVDLLGNAGLKGKT	5
STSPPSFSNV	0.04	DLLGNAGLKGKTNNH	14
SFSNVSENE	6.5		
NTNTSATSV	0.1	GLNVNIETGSRSRV	22
ENENTNTSAT	7.1	NIETGSRSRVNIGTG	66
TSATSVAPS	5.4		
NEQEKVDSI	0.29		
KKNMKSNEQ	13		
NPAIGKYEL	0.08		
STNLKNPAI	0.73		
FLKQKQDSF	0.01		
KSKSLFLKQK	0.04		
QNKQKSKSL	0.02		
NENDTDQNK	1.3		
LLGNAGLKGK	0.7		
GVDLLGNAGL	4.6		
LLGNAGLKGK	0.7		
GLKGKTNNH	1.7		
NVNIETGSR	0.34		
GLNVNIETG	12		
NIETGSRSR	0.88		
RSRVNIGTG	2		

**Table 3:** Pair of amino acid residues (highlighted by disulfide engineering) to be mutated.

Pairs of Amino Acid Residues	Chi3 Value	Energy in kcal/mol
Ile2-Lys5	−91.05	2.75
Phe9-Thr22	102.16	0.56
Val22-Gly21	−64.27	3.7
Ser16-Ala19	−64.37	6.37
His20-Pro23	−111.46	5.49
Thr36-Lue41	−109.49	7.37
Gln37-Val108	−86.81	2.82
Phe46-Arg56	−112.11	5.11
Tyr97-Ala101	−86.93	4.38
Asn111-Pro114	83.18	1.33
Gln136-Gly141	95.28	4.64
Gln149-Leu152	−111.28	3.38
Gly157-Thr163	−61.86	5.43
Gln162-Asp165	−101.08	4.65
Asn178-Pro184	−67.1	5.64
Ser203-Glu208	81.74	0.99
Lys204-Ile207	78.02	5.86
Thr218-Gly225	79.29	4.37
Pro224-Phe229	126.17	2.01
Gly225-Tyr228	118.82	3.29
Ser232-Gly239	125.75	3.07
Gly267-Asn278	121.18	5.67
Asp289-Pro294	101.78	2.12
Asp331-Gly339	−81.35	2.33
Gly358-Pro364	99.08	1.47
His376-Gly386	−82.67	5.08

**Table 4:** Top 10 refined model of vaccine with MHC-I obtained from FireDock.

Rank	Solution Number	Global Energy	Attractive VdW	Repulsive VdW	ACE	HB
1	6	−12.17	−8.14	1.36	−0.66	−1.44
2	7	3.70	−31.78	18.19	10.92	−5.88
3	4	15.71	−9.34	2.03	2.73	−1.13
4	1	20.24	−17.21	3.42	9.96	−0.90
5	3	21.66	−27.06	33.12	12.45	−2.59
6	9	77.86	−26.71	81.40	13.56	−5.32
7	2	164.85	−58.38	246.65	24.17	−8.25
8	8	257.49	−18.25	306.00	6.56	−4.93
9	5	1003.52	−46.18	1289.89	17.46	−6.73
10	10	2759.81	−34.85	3473.08	3.77	−5.51

**Table 5:** Top 10 refined model of vaccine with MHC-II obtained from FireDock.

Rank	Solution Number	Global Energy	Attractive VdW	Repulsive VdW	ACE	HB
1	9	−1.17	−7.24	1.98	1.78	−0.71
2	5	23.89	−3.24	0.00	0.80	−0.77
3	7	31.61	−0.77	0.00	1.39	0.00
4	3	241.30	−42.08	354.56	8.23	−4.07
5	2	460.31	−32.37	594.32	8.59	−4.83
6	6	473.83	−13.18	598.99	4.82	0.00
7	8	1395.95	−38.28	1774.07	16.30	−3.72
8	10	2536.69	−60.68	3213.80	16.12	−12.38
9	1	3964.79	−27.95	5027.40	−4.47	−5.94
10	4	3973.45	−52.13	5057.12	6.76	−7.93

**Table 6:** Top 10 refined model of vaccine with TLR-4 solution obtained from FireDock.

Rank	Solution Number	Global Energy	Attractive VdW	Repulsive VdW	ACE	HB
1	8	−4.52	−2.14	1.11	1.84	−1.11
2	5	3.89	−2.10	1.00	1.90	−0.77
3	7	1.61	−2.87	2.00	2.45	0.00
4	3	214.00	−41.00	381.00	9.16	−4.07
5	2	160.00	−35.08	594.32	8.59	−4.83
6	6	273.00	−17.10	598.99	4.82	0.00
7	9	335.00	−39.22	1774.07	16.30	−3.72
8	10	236.78	−61.69	3213.80	16.12	−12.38
9	1	961.10	-28.10	5027.40	−4.47	−5.94
10	4	978.36	-50.00	6059.10	8.18	−6.10

**Table 7:** Interactive residues of vaccine with MHC-I, MHC-II and TLR-4.

MHC-I-Vaccine Complex	MHC-II-Vaccine Complex	TLR-4-Vaccine Complex
ARG17	ARG4	PRO23
GLY16	THR3	GLU24
GLU19	PRO5	SER25
PRO15	ARG100	TYR26
ARG14	LYS126	ASP50
GLU89	GLU158	PRO49
ARG75	GLY125	SER76
VAL76	ARG189	ILE48
SER88	THR120	PHE74
THR86	PRO127	SER73
ALA139	ARG125	TYR72
THR142	ASP159	LEU69
MET138	ASP78	ALA97
THR73	TYR79	GLY96
GLU47	PRO81	ASP95
ARG45	LYS120	ILE93
LYS48	LYS121	GLY120
ASP38	GLY28	LEU117
	ASP29	GLN115
	GLU30	ILE114
	ARG93	PRO113
	ARG94	HIS148
	VAL160	ILE149
	ASN124	LYS150
	PHE7	ALA133
	THR87	LYS130
	HIS86	TYR65
	PHE87	PHE64
	ALA97	ALA107
	ASN98	ARG106

**Table 8:** MM PB/GB/ SA binding free energy analysis.

Energy Parameter	TLR-4-Vaccine Complex	MHC-I-Vaccine Complex	MHC-II-Vaccine Complex
**MM-GBSA**			
VDWAALS	−351.10	−310.51	−284.36
Electrostatic	−190.99	−139.02	−111.01
Delta G solv	25.30	49.74	36.00
Delta Total	−516.79	−399.79	−359.37
**MM-PBSA**			
VDWAALS	−351.10	−310.51	−284.36
EEL	−190.99	−139.02	−111.01
Delta G solv	26.32	47.10	39.17
Delta Total	−515.77	−402.43	−356.2

## Data Availability

The data presented in this study are available within the article.

## Supplementary Materials

The following are available online at https://www.mdpi.com/article/10.3390/ijerph191710742/s1, Table S1: Top 20 docked solutions of vaccine and MHC-I obtained from PATCHDOCK server, Table S2: Top 20 docked solutions of vaccine and MHC-II obtained from PATCHDOCK server, Table S3: Top 20 docked solutions of vaccine and TLR-4 obtained from PATCHDOCK server.
