# Supplementary material for: Novel Chimeric Vaccine Candidate Development against Leptotrichia buccalis
Source: Int J Environ Res Public Health. 2022 Aug 29;19(17):10742. doi: 10.3390/ijerph191710742 (PMC9518150; doi:10.3390/ijerph191710742)
Supplement: Supplementary file 1 [file ijerph-19-10742-s001.zip › ijerph-1792036-supplementary.pdf]

Table S1. Top 20 docked solutions of vaccine and MHC-I obtained from PATCHDOCK server. The solutions are ranked as per docking score. In the table, ACE is the atomic contact energy.

Solution No	Score	Area	ACE	Transformation
1	19262	2648.20	392.23	-2.52 0.35 1.59 37.40 -55.71 -37.09
2	18818	2398.60	488.08	0.69 0.30 -0.36 0.47 40.31 46.08
3	18664	2783.90	128.22	1.36 -0.70 0.21 -39.65 20.11 75.62
4	18330	2593.20	12.22	0.77 1.04 1.59 14.49 21.94 47.35
5	17254	2642.40	278.02	-0.20 -0.72 1.35 -8.93 -11.43 66.25
6	17216	2782.60	307.37	0.93 0.34 -0.25 -5.46 31.63 48.15
7	17168	2659.50	496.84	-2.55 -0.74 -1.61 -43.81 108.70 75.91
8	17148	2573.50	215.58	1.12 -0.89 2.93 49.53 2.93 98.67
9	17058	2311.60	359.63	-0.42 -0.81 1.15 -16.78 -1.81 68.51
10	16944	2584.20	265.83	1.94 0.64 -1.82 27.07 92.54 61.22
11	16842	2294.90	139.22	-2.61 -0.41 2.54 75.85 4.91 31.09
12	16838	2474.40	462.50	2.72 -0.59 0.54 -3.88 -32.91 68.06
13	16794	3164.10	344.40	1.25 -0.22 3.10 45.71 -13.47 52.82
14	16784	2971.90	-19.26	-2.32 -1.09 1.17 17.83 -21.05 -13.41
15	16752	2965.00	424.57	2.00 0.33 0.88 -38.50 -101.70 11.92
16	16748	2641.40	484.22	-3.00 0.42 -2.58 60.71 91.22 17.54
17	16738	2478.20	336.75	-2.48 -0.48 1.42 23.11 -62.30 -12.00
18	16672	2816.90	406.61	-0.32 -0.51 -2.41 54.73 10.54 59.84
19	16656	2406.10	80.53	1.20 1.08 -2.09 25.37 49.08 28.72
20	16618	2056.10	406.89	0.19 -0.45 -0.96 2.85 58.26 16.00

Table S2. Top 20 docked solutions of vaccine and MHC-II obtained from PATCHDOCK server. The solutions are ranked as per docking score. In the table, ACE is the atomic contact energy.

Solution No	Score	Area	ACE	Transformation
1	22148	3140.50	72.18	-1.06 0.79 -2.16 118.07 107.10 -76.91
2	20942	3097.70	224.01	-2.09 0.67 1.70 129.18 69.53 -64.43
3	20484	2587.30	188.46	-1.82 0.51 1.52 109.80 52.71 -64.59
4	19668	4061.10	363.57	-2.24 -0.16 2.55 194.71 50.98 -11.14
5	19032	3754.70	-10.82	-1.61 0.22 2.54 155.80 74.82 -40.62
6	18604	2719.10	297.63	0.09 -0.01 -1.43 142.25 139.09 -31.36
7	18470	2979.90	-20.72	-2.17 -0.69 -1.95 86.01 139.46 21.40
8	18256	2448.90	328.32	0.06 0.73 -1.07 164.73 89.79 -12.07
9	18210	3632.10	-101.67	-2.12 -0.45 1.36 110.86 25.49 1.57
10	17224	3549.80	429.31	3.00 -1.51 -2.11 72.85 59.68 43.56
11	17060	2263.10	287.00	3.04 1.19 -2.00 65.68 127.56 13.44
12	17034	2453.20	339.36	1.74 -0.82 -0.78 88.75 88.05 81.93
13	17008	2556.00	276.98	1.19 0.43 1.06 38.33 30.46 3.29
14	16744	3265.10	-251.28	-1.74 -0.74 -1.69 91.31 142.27 14.61
15	16398	3126.90	-77.10	-1.26 0.95 -2.34 99.15 99.38 -80.30
16	16282	3598.20	251.66	-2.52 0.03 -1.97 106.95 110.24 -18.29
17	16222	2635.10	332.61	3.06 -0.50 1.64 161.45 21.85 33.60
18	16220	2160.00	303.70	-3.00 0.08 2.69 178.71 90.45 -8.86
19	16178	2732.90	357.21	2.09 0.04 1.39 145.08 6.45 50.75
20	16150	2626.10	53.88	-1.69 0.63 1.40 97.94 64.02 -74.42

Table S3. Top 20 docked solutions of vaccine and TLR-4 obtained from PATCHDOCK server. The solutions are ranked as per docking score. In the table, ACE is the atomic contact energy.

Solution No	Score	Area	ACE	Transformation
1	25398	4819.20	361.95	-0.83 0.59 -0.05 -62.63 9.72 -60.32
2	22582	3139.00	388.72	-0.90 0.57 0.04 -65.77 3.90 -57.50
3	19442	3353.20	125.58	1.59 1.16 1.82 7.55 -45.05 -102.59
4	19292	2847.30	107.04	1.24 1.19 2.21 20.06 -37.38 -108.00
5	18032	2590.80	432.37	2.43 -0.14 2.81 -33.25 33.45 3.91
6	17924	2850.10	458.75	0.59 1.00 0.18 -93.08 13.00 -39.17
7	17702	3485.10	345.96	1.56 1.34 1.79 -0.77 -30.67 -113.46
8	17292	2745.30	346.02	0.27 -0.49 -2.70 90.14 15.40 -16.35
9	17096	3720.90	18.04	0.88 0.09 -0.11 -13.18 13.14 13.91
10	17066	3101.40	77.31	0.44 0.01 1.43 -46.56 -50.71 -2.30
11	16928	2216.10	477.71	2.78 -0.25 2.72 40.94 -2.21 -32.96
12	16780	2792.30	211.54	-2.44 -0.37 -2.06 -2.04 137.36 -45.66
13	16664	3325.00	324.35	2.81 0.62 2.53 34.89 -27.23 -20.69
14	16634	2639.40	430.77	-2.02 -0.30 2.41 19.63 -30.53 -16.00
15	16446	2868.40	293.90	1.33 -0.79 2.69 -66.25 13.22 -26.58
16	16442	4321.80	418.21	-0.92 0.53 -0.12 -62.94 6.71 -59.02
17	16336	3738.60	447.77	-2.59 0.48 -1.41 -60.17 50.61 -46.66
18	16200	2740.90	238.73	0.98 1.35 2.25 5.53 -24.85 -117.40
19	16186	2794.50	89.17	-1.75 0.06 1.62 19.63 -6.02 -99.64
20	16174	2797.40	409.05	2.01 -0.50 0.46 -44.82 -39.20 -17.03
